# Supplementary material for: Management of Non-Communicable Diseases in Kosovo: A Scoping Review
Source: Int J Environ Res Public Health. 2023 Feb 13;20(4):3299. doi: 10.3390/ijerph20043299 (PMC9961581; doi:10.3390/ijerph20043299)
Supplement: Supplementary file 1 [file ijerph-20-03299-s001.zip › ijerph-2161792-supplementary.pdf]

Supplementary Materials

Supplementary Materials S1 - Search strategy

PubMed

Link: https://www.ncbi.nlm.nih.gov/pubmed/

Date Searched: 26 December 2021

Number of Results: 114

Search update: 02 February 2023

Number of results: 58

Search strategy: diabetes OR "diabetes melitus" OR hypertension OR "high blood pressure" OR "breast cancer" OR "cervical cancer" OR "non communicable diseases" OR NCD OR NCDs OR NCD-s AND Kosovo

Scopus

Link: https://www-scopus-com.dartmouth.idm.oclc.org/search/form.uri?display=basic

Date Searched: 26 December 2021

Number of Results: 143

Search update: 02 February 2023

Number of results: 12

Search strategy: TITLE-ABS-KEY ((diabetes OR "diabetes melitus" OR hypertension OR "high blood pressure" OR "breast cancer" OR "cervical cancer" OR "non communicable diseases" OR NCD OR NCDs OR NCD-s) AND (Kosovo))

Web of Science

Link:http://apps.webofknowledge.com.dartmouth.idm.oclc.org/WOS_GeneralSearch_input.do?product=WOS&search_mode=GeneralSearch&SID=6FMLm2qZ9JVV1wTRJk5&preferencesSaved=

Date Searched: 26 December 2021

Number of Results: 113

Search update: 02 February 2023

Number of results: 10

Search strategy: (AB=(diabetes) OR AB=(diabetes melitus) OR AB=(hypertension) OR AB=(high blood pressure) OR AB=(breast cancer) OR AB=(cervical cancer) OR AB=(non communicable diseases) OR AB=(NCD) OR AB=(NCDs) OR AB=(NCD-s)) AND (AB=(Kosovo))

Google Scholar (English language search)

Link: https://scholar.google.com/

Date Searched: 26 December 2021

Number of Results: 200

Search update: 02 February 2023

Number of results: 50

Search strategy: diabetes OR "diabetes melitus" OR hypertension OR "high blood pressure" OR "breast cancer" OR "cervical cancer" OR "non communicable diseases" OR NCD OR NCDs OR NCD-s AND Kosovo

Google Scholar (Albanian language search)

Link: https://scholar.google.com/

Date Searched: 26 December 2021

Number of Results: 200

Search update: 02 February 2023

Number of results: 50

Search strategy: diabeti OR hipertensioni OR “tensioni i lartë” OR “kanceri i gjirit” OR “kanceri i qafës së mitrës” OR “sëmundjet jo të transmetueshme” OR “sëmundjet kronike”

Table S1 - Study characteristics and findings

Author	Year	Type of publication	Factors influencing the management of NCDs (Subthemes reported)	Information on the management of NCDs (Subthemes reported)	Outcomes of management of NCDs (Subthemes reported)	NCDs reported	Key findings
Barbullushi et al. [21]	2002	Journal article		Treatment, clinical pathways, application of clinical guidelines		Overall	✓ Successful long-term treatment and management of NCDs in Kosovo will highly depend on the transfer of knowledge, training of staff, economic feasibility, development of clinical pathways, and application of protocols.
Haxhikadrija et al. [22]	2008	Journal article			NCD-related mortality rate	Breast cancer	✓ The global incidence of breast cancer is rising, and mortality is rising in places without early detection systems, such as Kosovo.
Knowles et al. [23]	2008	Report	Supply and capacity of healthcare staff	Diagnosis, treatment, clinical pathways, application of clinical guidelines	NCD-related burden of disease	Breast cancer, cervical cancer	✓ The staff is usually unskilled or undertrained in properly using equipment for cervical and breast cancer screening.<br>✓ Diagnostic support is frequently unavailable or challenging to obtain at the point of the presentation, necessitating the use of private facilities, often in Prishtina (capital) rather than locally.<br>✓ The UCCK breast and cervical cancer units are unable to provide a comprehensive tertiary clinical service to Kosovar women.<br>✓ From the initial presentation to the final therapy, the patient's journey is challenging and ineffective. Self-referral to secondary or tertiary clinics persists.<br>✓ When it comes to breast cancer, there is widespread evidence of the insufficient investigation, decision-making that is not based on best medical practice standards, and inappropriate treatment and follow-up by providers and patients.<br>✓ UCCK is unable to provide crucial oversight of clinical routes, training, and ongoing professional development for staff working in the field of breast and cervical cancer in Kosovo.<br>✓ Clinical decisions regarding secondary care therapy do not follow any established set of local or international criteria, and they differ significantly amongst healthcare facilities.<br>✓ Women with cancer, without basic medical or social assistance or support, bear a hidden weight of disease.
Schneider et al. [24]	2008	Report	Funding of healthcare	Referral in private sector		Overall	✓ Patients seek care in the private sector and purchase drugs from pharmacies due to public health facilities' shortage of medicines and resources.<br>✓ The current benefit package is intended to cover all public health facilities and a restricted treatment package when traveling overseas. In reality, more than 80% of pharmaceuticals purchased by Kosovo patients are paid out of their pocket, owing to a

							limited, government-run program for vital drugs that is underfunded and plagued with logistical issues.
Ajvazi et al. [25]	2009	Journal article	Availability of clinical guidelines			Diabetes	✓ There are no specific treatment protocols for complications or other important aspects of diabetes treatment and control.
Balkan Investigative Reporting Network [26]	2009	Report	Medications and medical supplies	Referral in private sector		Overall	✓ Media monitoring has revealed that following the first visit to a public health facility, doctors and employees directed patients to private clinics run by themselves, their colleagues, or contacts.<br>✓ Other data confirmed a relationship between public health workers and privately operated clinics.<br>✓ Insulin for diabetic patients is supposed to be covered by the state.
Devolli-Disha et al. [27]	2009	Journal article		Diagnosis		Breast cancer	✓ Ultrasound is available for breast cancer diagnosis.
FRIDOM [28]	2009	Report	Medications and medical supplies, availability of clinical guidelines	Referral in private sector		Overall	✓ Hospitals have long-standing problems accessing necessary drugs from the essential list. This has left the patients to pay for the drugs themselves in the past.<br>✓ In recent times, there have been significant improvements in the supply of drugs to health institutions.<br>✓ There has been limited progress in the development of clinical protocols.<br>✓ Progress in the development of treatment protocols has been made thanks to external technical support.<br>✓ Physicians engaging in dual practice and referring patients to their private practice are driving an increase in demand for private-sector care.<br>✓ To meet consumer demand for private-sector treatment, the number of private providers has continuously increased in Kosovo, particularly in urban areas, with an estimated 30% of primary care visits taking place in private facilities.
Burkle [29]	2010	Journal article			NCD-related mortality rate	Overall	✓ Over half of the hospital deaths are caused by NCDs.
Percival et al. [30]	2010	Journal article			NCD-related mortality rate	Overall	✓ Over half of the hospital deaths are caused by NCDs.<br>✓ Cardiovascular, renal and lung illness, chronic back pain, and ulcer/gastritis are the most common adult health disorders.
Bakalli et al. [31]	2011	Journal article			NCD-related mortality rate	Hypertension	✓ Arterial hypertension, accompanied by atherosclerosis, is the leading cause of death.
Begolli et al. [32]	2011	Report		Diagnosis, referral among levels of care, referral in private sector		Overall	✓ Besides late diagnosis, another important issue is the uncertainty of whether the therapy will be successful for the patient.<br>✓ Regulation of referral where noncompliance should have financial implications has yet to occur.<br>✓ There are cases of referral in the private sector to use unnecessary diagnostic services.

Bielecka-Dabrowa et al. [33]	2011	Journal article		Diagnosis, treatment, application of clinical guidelines		Hypertension	✓ Hypertension diagnosis depends on population awareness of hypertension risks/danger, preventive care, and medical interventions.<br>✓ It is questionable to what extent the management of hypertension is done via a holistic approach in terms of management of cardiovascular risk and hypertensive therapeutic personalization.<br>✓ Medical compliance with hypertension treatment guidelines varies across Eastern Europe and the Western Balkan countries, including Kosovo.
Daullxhiu et al. [34]	2011	Journal article			NCD-related mortality rate	Hypertension	✓ Hypertension is one of the independent predictors of poor exercise capacity for chronic stable heart failure patients, which often leads to high mortality rates.
Health for All [35]	2011	Report			Access to care	Overall	✓ The Roma, Ashkali, and Egyptian communities have serious issues related to healthcare access.
O'Hanlon et al. [36]	2011	Journal article	Supply and capacity of healthcare staff			Overall	✓ The capacities and clinical competence of medical staff is another domain where advancement is needed to increase their skills and capacities to treat patients with NCDs.
Balkan Investigative Reporting Network [37]	2012	Report	Medications and medical supplies	Referral among levels of care		Overall, diabetes	✓ Ministry of Health has often failed to provide the essential list of medicines to Kosovo inhabitants, including medicines for diabetes.<br>✓ Most primary care institutions have to purchase medications from their funds, including hypodermic needles and syringes.<br>✓ The primary care institutions are under-supplied, and sometimes are given drugs that they do not require or that are expired.<br>✓ There are cases when pharmaceuticals, particularly insulin, are diverted from primary care institutions and sold to private pharmacies due to a lack of competent control in the distribution and management of supplies.<br>✓ An important contributor to the lack of coordination between different levels of healthcare for NCDs is the lack of a functional Health Information System.
Luta et al. [38]	2012	Journal article			Access to care	Overall	✓ Women and men from Roma, Ashkali, Egyptian, and Gorani communities confront the greatest geographic, socioeconomic, and cultural hurdles to healthcare.
Ministry of Health [39]	2012	Report		Diagnosis, treatment		Overall	✓ Kosovo's public health care system provides only basic diagnostics and treatment of NCDs.
Arifi et al. [40]	2013	Journal article			NCD-related mortality rate	Diabetes	✓ The hospital mortality rate for necrotizing fasciitis in Kosovo from 2005 to 2010 was 22.6%. All fatal cases suffered from diabetes.
Hee Lee-Kwan et al. [41]	2013	Report	Funding of healthcare	Referral in private sector		Overall	✓ Patients with NCDs, such as hypertension and diabetes, are required to acquire the drugs themselves, which cost 40 to 60 Euros per month on average.

							✓ Some patients decided to seek treatment directly at private hospitals when their symptoms were severe and were not referred by the public institution.<br>✓ Patients who go directly to a private hospital usually have a good understanding of their symptoms and believe that going to a private hospital gets the "job done" efficiently (both in terms of time and money) without the difficulties that can occur at a public hospital. When cost becomes a problem, however, patients turn to public institutions.
Hoxha [42]	2013	Report	Medicines and supplies, supply and capacity of healthcare staff, funding of healthcare	Treatment, counseling services	Satisfaction with care	Overall, diabetes, breast cancer, cervical cancer	✓ Kosovo's healthcare system is underfunded, which is reflected in the lack of medical supplies and treatment for breast and cervical cancer in institutions.<br>✓ There have been several efforts by the primary care institutions in Kosovo to develop capacities for diabetes treatment, but there is still a long way to go before any improvements become visible.<br>✓ There is a lack of competent medical staff, especially nurses, who can counsel type 2 diabetes patients.<br>✓ Usually, patients first visit and talk with a family doctor about certain NCDs. However, some patients go directly to a specialist in primary healthcare or private institutions, the UCCK, or other regional hospitals.<br>✓ The consulting doctor is either a family doctor or a specialist in the public or private sectors.<br>✓ Patients with breast and cervical cancer seem to appreciate the professionalism of the medical staff at the secondary and tertiary care level, although physicians are more critical of themselves.
Balidemaj et al. [43]	2014	Journal article	Supply and capacity of healthcare staff	Treatment		Overall	✓ Kosovo government has decided to contribute two million Euros to a program that assists patients in obtaining unique therapies (i.e., advanced cancer care) that are not available in Kosovo.<br>✓ Kosovo has one of the lowest supply of healthcare staff in Europe. The immigration of health personnel from Kosovo is still increasing and has aggravated the situation regarding the supply of medical staff. As a result, there is an urgent need to develop strategies that will minimize the harm of the movement of health personnel and will maintain a proper level of health services provided to the patients.
Bhabha et al. [44]	2014	Report			Access to care	Overall	✓ The Roma, Ashkali, and Egyptian communities have serious issues related to healthcare access.<br>✓ There is limited access for patients living in poverty, the elderly, people with disabilities, and those living in remote areas, who typically have trouble accessing health services.
Tahiri et al. [45]	2014	Journal article			Satisfaction with care	Overall	✓ Roughly half of the patients receiving primary care are satisfied with the overall quality of medical services, the relationship

							between doctor and patient, and the organization of care for NCDs.<br>✓ Patients who are younger, from urban areas, and employed were much more satisfied with the overall quality of their health encounters.
Uka [46]	2014	Report	Funding of healthcare			Overall	✓ Another important barrier to care is that the healthcare staff and patients in Kosovo declare that informal payment for NCDs exists in the public healthcare sector in Kosovo.<br>✓ The frequency of informal payments differs depending on the types of medical services.<br>✓ Patients, contrary to healthcare professionals, believe that informal payments are more common.
Vian [47]	2014	Report	Medications and medical supplies, funding of healthcare			Overall, breast cancer, cervical cancer	✓ In total, the Kosovo government spends about 21.7 million Euros on medicines a year, excluding cancer medications.<br>✓ It seems that the current level of funding is inadequate to cover the needs for treating breast and cervical cancers.
Davies et al. [48]	2015	Report	Supply and capacity of healthcare staff, availability of clinical guidelines	Diagnosis, treatment, clinical pathways, application of clinical guidelines		Breast cancer, cervical cancer	✓ Supply issues manifest in the provision of care related to NCDs. For example, there is no efficient screening for breast and cervical cancer.<br>✓ There are trained clinicians but retaining these abilities needs ongoing exposure to a volume of screenings (i.e., mammograms) substantially greater than what is currently done at UCCK.<br>✓ There are no national clinical guidelines or Standard Operating Procedures for any aspect of breast or cervical screening, follow-up, or treatment.<br>✓ Clinical guidelines from other countries can be downloaded from the internet, but the quality of these and their relevance to health care as practiced in Kosovo is not automatic.<br>✓ More recently, the protocol for screening and treatment of precancerous cervix (cervix uteri) damages has been approved.<br>✓ International stakeholders have encouraged and supported the use of guidelines when it comes to cervical screening. Some serious effort has been made by governmental and non-governmental institutions with the support of international actors to implement them across the country.<br>✓ There is still a large proportion of breast cancer cases that are diagnosed in their late stages, which is mainly due to the absence of effective breast cancer screening programs.<br>✓ Currently, there are several methods or tests available that are commonly used for breast cancer screening, including mammography, breast self-examination, and clinical breast examination.

							✓ Most cervical cancer cases in Kosovo are usually diagnosed in the late stages, suggesting that the majority of women are more likely to have invasive cancer at the onset.<br>✓ Screening for cervical cancer is limited, as is the quality of the services, the coordination of care, the proper follow-up of screened people, and the establishment of a definitive diagnosis and treatment.<br>✓ Patients might be lost to any form of follow-up in the absence of a defined pathway, and there appears to be little motivation for service providers to assume ownership of a patient flow from the point of referral through an acceptable and documented outcome.<br>✓ The lack of a defined patient pathway for cases of suspected breast or cervical cancer exemplifies the problems related to the management of NCDs in the country.
Hoxha et al. [49]	2015	Policy brief	Medications and medical supplies, funding of healthcare	Treatment, counseling services, referral in private sector		Diabetes	✓ People turn to private health clinics, mainly for their children and the elderly, when they require special treatment for their diseases. Individuals, i.e., diabetes patients, spend a significant portion of their income on complications care. In 2013, the average annual health costs per diabetic patient were 830 Euros.<br>✓ Patients with diabetes in Kosovo are treated at all three levels of the healthcare system. Patients are not required to pay for the required tests (e.g., oral glucose, tolerance, and blood tests) and treatments if they visit public care facilities. For those tests, the public health institutions practice official co-payments, which cost only a few euros. However, if patients receive the treatment in private facilities, they are obliged to pay.<br>✓ Insulin does not appear to represent a significant portion of the cost, with patients reporting an average monthly cost of only 3.31 Euros since insulin is usually provided free of charge in public hospitals.<br>✓ Medicines for oral administration are the most commonly utilized therapy for diabetes.<br>✓ The availability of important diabetes medications should be greatly enhanced, medical devices should be provided to public institutions in sufficient quantities, and free test tapes for monitoring glucose levels should be provided.
Hoxha et al. [50]	2015	Policy brief		Treatment	Access to care, satisfaction with care	Diabetes	✓ Patients with diabetes have access to general and specialized care.<br>✓ Around 59% of people with diabetes visit a family doctor, about 70% visit an internal medicine specialist and/or a diabetes specialist, and about 39% visit an endocrinologist.<br>✓ Diabetic patients are most satisfied with visits to diabetes specialists and endocrinologists.

							✓ Visits to other health providers, such as family doctors and internists, tend to leave them unsatisfied.
Hoxha et al. [51]	2015	Report	Medications and medical supplies, funding of healthcare	Referral in private sector		Overall	✓ Besides referral to seek healthcare services in the private sector, there are also cases of referral by family doctors to buy presumably inadequate or unnecessary prescribed medicines in certain pharmacies.<br>✓ There is a confirmed absence of asthma inhalers, hypertension medicines, diabetes medicines, intramuscular and intravenous supplies, medicines to treat high blood cholesterol, syringes, and insulin. As a result, patients must often acquire medication supplies on their own.
Raunio et al. [52]	2015	Policy brief	Funding of healthcare		Access to care	Overall	✓ In the case of Roma, Ashkali, and Egyptian communities, discrimination occurs due to poverty and inability to pay for treatment, medication, transportation, or informal payments to health practitioners, lack of identification documents, and hence ineligibility to use public healthcare providers. There is also discrimination based on ethnicity.
Raunio et al. [53]	2015	Policy brief			Access to care	Overall	✓ The difficulty in paying for medical treatment was cited by 55.1% of Roma, Ashkali, and Egyptians as the reason for not visiting a doctor more frequently.
Dixit et al. [54]	2016	Report		Counseling services	Access to care	Breast cancer	✓ Kosovo's health system has yet to develop a capacity to support middle-aged and elderly women between 50 and 69 years old and provide adequate counseling services in case of diagnosis and detection of breast cancer.<br>✓ Women and men from Roma, Ashkali, Egyptian, and Gorani communities confront the greatest geographic, socioeconomic, and cultural hurdles to healthcare.<br>✓ When it comes to breast and cervical cancer, with the exception of Serbs, the minority ethnic groups have a harder time getting care than the Albanian majority.
Farnsworth et al. [55]	2016	Report		Diagnosis	Access to care	Hypertension, breast cancer	✓ Kosovars are using specific healthcare services, such as screenings and general health exams, to detect hypertension risk factors early enough to enable treatment and avert danger. For example, healthcare providers use the measuring of blood pressure, blood tests, urine samples, and physical exams of patients to detect hypertension risk factors.<br>✓ Important investments for the diagnosis of breast cancer have been made. Only by 2015, six mammography units were provided, including a mobile one, which has been used in different locations across the country.<br>✓ The availability of cervical cancer screenings is a situation of mismatch in supply and demand of the health staff.

							✓ It appears that most minority ethnic groups have a worse time getting medical treatment than the Albanian majority.<br>✓ The Bosnian, Roma, Ashkali, Egyptian, and Gorani communities faced the greatest economic impediments, while Serbs fared better.<br>✓ Goranis had the highest geographic impediments to healthcare, including the lack of emergency services, due to their distant position.
Giordano et al. [56]	2016	Journal article		Diagnosis		Breast cancer	✓ Kosovo lacks information about breast cancer and disease staging.
Kantar TNS [57]	2016	Report	Availability of clinical guidelines	Treatment, referral in private sector		Diabetes, hypertension	✓ External assistance has made available clinical guidelines.<br>✓ 35% of respondents who know they have diabetes have stated that they had their blood sugar monitored by a doctor or other health practitioners.<br>✓ Aside from going to a primary healthcare center, most respondents (53%) measure their blood pressure on their own, 40% do it with the help of a neighbor or family member, and in 27% of cases, they go to their regional hospital.<br>✓ When patients' blood pressure rises, 56% take antihypertensive medication, 22% visit a doctor, and 12% restrict their diet. 28% of respondents said they are unsure what to do if their blood pressure increases.<br>✓ Users of healthcare services are likely to have their blood sugar tested twice as often as non-users.<br>✓ In the private sector, besides the higher prices for services, doctors sometimes offer services in medical fields that they are not specialized.
Moore et al. [58]	2016	Journal article	Availability of clinical guidelines			Overall	✓ Some guidelines have remained in the planning stages. One of the reasons for this is that stakeholders lack awareness and/or knowledge of clinical guidelines.
Thompson et al. [59]	2016	Journal article	Availability of clinical guidelines	Counseling services		Hypertension	✓ In partnership with external institutions, Kosovar nurses worked together on a two-year project to rebuild primary health care in Gjilan. In this model of care, nurses were considered a focal point for interaction with the patients. The project invested in training them to gain new skills, especially learning how to teach patients about lifestyle changes associated with controlling hypertension.
Ramadani et al. [60]	2016	Journal article			NCD-related mortality rate	Other	✓ The mortality rate for pancreatic cancer was 17.2 per 100,000 people.
Zahorka et al. [61]	2016	Report		Diagnosis, treatment		Hypertension	✓ Assessment of the quality of clinical examinations reveals shortcomings in the assessment and examination of patients. For example, incomplete anamnesis, physician examination, lack of proper recording of findings for individual patients, etc.

							✓ Issues relating to the hazards of untreated disease were less frequently explained to patients.
Zahorka et al. [62]	2016	Report		Referral among levels of care		Overall	✓ While patient referral exists as a formal process, counter-referral (feedback from secondary or specialist to primary care) is not generally performed, which adds to the fragmentation of care.
Hoxha et al. [63]	2017	Policy brief		Referral among levels of care		Overall	✓ The establishment of Kosovo Hospital and University Clinical Services (KHUCS) aimed to integrate the hospital healthcare system (UCCK and regional hospitals) along professional lines. It was also envisioned that this system would coordinate specialized health services and ensure a high level of exchange of knowledge and professional experience between the constituent institutions.
Hoxha [64]	2017	Report		Referral in private sector		Overall	✓ Unfortunately, because state-of-the-art treatment is not readily available in the country, frequent referrals to neighboring countries are necessary.<br>✓ Although protocols are not widely applied, there is motivation and will to apply them.
Hoxha et al. [65]	2017	Policy brief	Funding of healthcare			Overall, diabetes	✓ With the establishment and functionalization of the Health Insurance Fund, will be covered all laboratory tests and some medicines for chronic diseases, such as diabetes.
Hoxha et al. [66]	2017	Policy brief			Satisfaction with care	Overall	✓ Healthcare providers are dissatisfied with the availability of pharmaceuticals and health products, medical equipment, public-private sector collaboration, and the remuneration of health professionals at their institutions.
Hughes et al. [67]	2017	Journal article		Treatment, clinical pathways		Diabetes	✓ The pharmacists seem to also play a role in offering services for extended diabetes patients.<br>✓ As the desired result of implementing this integrated care mode, every diabetic patient's care will begin with a thorough examination at the time of diagnosis, which will include a review of diabetes problems and risk factors for complications. This serves as the foundation for ongoing care and interaction, which includes the development of a treatment plan, treatment administration, monitoring, and review.
Bajraktari et al. [68]	2018	Journal article		Treatment	NCD-related mortality rate	Hypertension	✓ Hypertension management has moved from one-off readings to average 24-hour monitoring.<br>✓ In the general population of Kosovo, arterial hypertension is one of the major risk factors for mortality and morbidity.
Jakupi et al. [69]	2018	Journal article	Medications and medical supplies, funding of healthcare			Other	✓ The use of anti-cancer drugs in Kosovo has varied significantly over time. This is a cause for concern. Anti-cancer medications cost 16.49 million Euros between 2011 and 2013.

Zejnullahu-Raci et al. [70]	2018	Journal article		Diagnosis, treatment	NCD-related mortality rate	Cervical cancer	✓ HPV-based cervical cancer screening and vaccination may avoid more than 70% of cervical precancerous lesions in the case of Kosovo. Hence it is critical to implement both programs as soon as feasible in the national healthcare system.<br>✓ No accurate statistics on cervical cancer incidence and death, as well as high-risk Human Papilloma Virus (HPV) prevalence and HPV type distribution, are available in Kosovo.
Bytyci et al. [71]	2019	Journal article		Counseling services, clinical pathways		Diabetes	✓ Via external aid, primary care institutions in 20 municipalities have been establishing motivational counseling services for type 2 diabetes mellitus.<br>✓ With external assistance, an integrated care model for patients 65 years and older with type 2 diabetes mellitus. As a result, individual care plans are developed for patients 65 years and older.
Bytyqi-Damoni et al. [72]	2019	Journal article		Treatment		Diabetes	✓ There is a record of the availability of comprehensive care, such as regulating postprandial hyperglycemia by blocking the a-glycosidase enzyme in the digestive tract, which prolongs overall carbohydrate digestion.<br>✓ Several commercially available antidiabetic medications, such as metformin, acarbose, voglibose, and miglitol, are being used to treat a-amylase and a-glycosidase enzyme suppression in diabetes mellitus.
Cuperjani et al. [73]	2019	Journal article		Treatment		Breast cancer	✓ Depending on the size or location of the lesion, as well as the existence of an extensive intraductal component, about one-third of women with breast cancer in Kosovo still have a mastectomy.
World Health Organization [74]	2019	Report	Availability of clinical guidelines	Application of clinical guidelines	NCD-related hospitalization rate	Diabetes, hypertension	✓ There are two official protocols for pharmaceutical and non-pharmaceutical treatment of diabetes that have been approved by the Ministry of Health.<br>✓ The application of protocols has varied. Nevertheless, there have been serious efforts to implement them in primary care.<br>✓ More recently, with support from external projects, protocols for the management of hypertension have been designed and approved by the Ministry of Health and are being applied in primary care in 20 municipalities.<br>✓ Overall, the number of diabetic and hypertensive hospitalizations in Kosovo has been increasing.<br>✓ Between 2012 to 2014, there was a slight decline.<br>✓ Since 2014, diabetes hospitalizations have been more constant, with a minor increase for both genders in 2016.<br>✓ Both genders have hospitalization rates of 776 for hypertension and 396 for diabetes per 100,000 people. This is higher than the OECD average of 33 nations in 2015.

Ymerhalili et al. [75]	2019	Journal article		Clinical pathways		Diabetes, hypertension	✓ The Municipality of Fushe Kosova was the first municipality to test an integrated care model, which also included better coordination of health and social sector services.
Dimitrova et al. [76]	2020	Journal article	Availability of clinical guidelines			Overall	✓ Incorporation of the European Society of Medical Oncology's suggestions into national guidelines is limited.
Eyvazzadeh et al. [77]	2021	Report		Referral among levels of care		Overall, breast cancer	✓ The existing referral mechanism for breast cancer, and highly likely for any NCD, is inefficient.<br>✓ Patients frequently forego primary care in favor of secondary or tertiary care, although primary care facilities can provide the required services.<br>✓ The system currently only connects a few primary and secondary clinics, and there is no way to send electronic referrals or follow up with patients at the tertiary level in UCCK.
Milosevic et al. [78]	2021	Journal article			Satisfaction with care	Overall	✓ Among ethnic Serb communities, the presence of NCDs was connected with a lower level of self-rated health status. A shorter waiting time and appropriate finances were linked to a higher level of satisfaction with healthcare (57.8%).
Podvorica et al. [79]	2021	Journal article		Counseling services		Other	✓ The role of nursing education of patients with heart disease was crucial and resulted in increased knowledge of self-care for the disease.
Bytyci Katanolli et al. [80]	2022	Journal article		Counseling services		Overall	✓ Counselling could best tackle individual barriers such as health-related problems.
Bytyci Katanolli et al. [81]	2022	Journal article		Counseling services		Overall	✓ 22% of the eligible patients received counseling services in the form of motivational counseling.
Ejupi et al. [82]	2022	Journal article		Counseling services, treatment		Breast cancer	✓ Single chemotherapeutic agent most frequently used was herceptin (40%), followed by anastrazole (30%) and tamoxifen (30%), respectively.<br>✓ Frequencies of combination therapies used were as follows: 25.99% FEC (fluorouracil + epirubicin hydrochloride + cyclophosphamide), 18.52 % AC-T (doxorubicin hydrochloride + cyclophosphamide + palitrixel), 18.52% CMF (cyclophosphamide + methotrexate + fluorouracil), 14.8% CAF (cyclophosphamide + doxorubicin hydrochloride + fluorouracil), 14.81% TAC (docetexel + doxorubicin hydrochloride + cyclophosphamide), and 7.41% AC (doxorubicin hydrochloride + cyclophosphamide), respectively.<br>✓ To overcome side effects patients, in 50% of cases consult doctors,
Hoxha et al. [83]	2022	Journal article		Treatment		Breast cancer	✓ The surgical treatments for breast cancer have changed dramatically, from radical mastectomy to breast-conserving surgery.
Hoxha et al. [84]	2022	Journal article	Application of clinical guidelines			Other	✓ There are often odd and wide variations in clinical signs and examination influencing the decision for heart failure diagnosis.

Obas et al. [85]	2022	Journal article		Diagnosis, treatment		Diabetes, hypertension, other	✓ Cases remain undetected for hypertension (19%), diabetes (16%) and Chronic Obstructive Pulmonary Disease (COPD) (45%).<br>✓ Uncontrolled hypertension (28%), diabetes (79%), and COPD (76%) are frequent.<br>✓ There is substantial need for improving NCD prevention and control along the system of care.

NCD = Non Communicable Disease, UCCK = University Clinical Center of Kosovo
